# Can the balance evaluation systems test be used to identify system-specific postural control impairments in older adults with chronic neck pain?

**DOI:** 10.3389/fmed.2022.1012880

**Published:** 2022-10-28

**Authors:** Thanya Madsalae, Tanapat Thongprong, Chatchada Chinkulprasert, Rumpa Boonsinsukh

**Affiliations:** Division of Physical Therapy, Faculty of Physical Therapy, Srinakharinwirot University, Nakhon Nayok, Thailand

**Keywords:** elderly, geriatrics, fall, clinical scale, assessment

## Abstract

**Background:**

Older adults with chronic neck pain (CNP) demonstrate impaired postural control. The Balance Evaluation Systems Test (BESTest) is used to assess systems underlying postural control impairments, but its use in CNP has not been reported. This study assessed whether the BESTest can identify postural control impairments in CNP as well as the level of BESTest item difficulty by Rasch analysis.

**Materials and methods:**

This cross-sectional study recruited thirty young adults (YOUNG) aged 20–40 years and eighty older adults aged 60 years or older [without neck pain (OLD) = 60, with chronic neck pain (CNP) = 20]. Questionnaires were administered to collect demographic data, intensity of neck pain (VAS), patient’s self-rated neck pain and disability (NDI), and balance confidence in daily activities (ABC). The BESTest was used to assess postural control.

**Results:**

The CNP group showed the lowest ABC scores. Compared to the YOUNG group, the BESTest score was significantly lower in the OLD group, while the CNP group showed the lowest score, suggesting that balance control deteriorated from the normal aging process and further declined in the CNP group, especially in biomechanical constraints, transitions–anticipatory postural adjustment, and reactive postural response (*p* < 0.05). Using scores from these three sections, the BESTest was accurate at the cutoff score of 48.5 out of 51 for differentiating the older adults whose daily life are affected by neck problems (using the NDI as a reference) with a high AUC (0.79), sensitivity (72%), and specificity (69%). The Rasch analysis revealed that the Timed Up and Go with dual task test was the most difficult BESTest item for all groups, whereas 14 items showed more difficulty for the CNP group.

**Conclusion:**

The BESTest can be used to identify postural control impairments in CNP patients, even those with moderate pain and mild disability with a high level of physical functioning. The combined score of biomechanical constraints, transitions–anticipatory postural adjustment, and reactive postural response domains was suggested for the detection of older adults whose daily lives are affected by neck problems. This will also help clinicians consider the management of neck pain to prevent falls in CNP.

## Introduction

Falls are a major public health problem globally. People aged 60 years or older suffer the greatest number of fatal falls leading to unintentional injury or death (1). The incidence of falls in older adults is increased by age-related declines in the systems responsible for controlling balance, also known as the postural control system (2). This includes declines in the musculoskeletal system, internal representations, adaptive mechanisms, anticipatory mechanisms, sensory strategies, individual sensory systems, and neuromuscular synergies (3).

Among chronic musculoskeletal conditions, neck pain is one of the most common complaints in the elderly population and ranks as the fourth leading cause of disability worldwide (4). Much evidence has confirmed that older adults with chronic neck pain (CNP) demonstrate a fear of falling, decreased physical performance and increased risk of falls more than older adults without CNP (5–8). In addition to decreased mobility of the cervical joint (9) and muscle strength (10), older adults with CNP also demonstrate a decrease in sensorimotor integration presented by reduced gait speed, impaired postural control, and cervical position sense (8). Alterations in sensory integration can be caused by pain (11, 12), inflammatory events (13), awkward postures (14), static and repetitive work, or trauma (15) that affect the sensitivity of the cervical joint and muscle receptors in both supraspinal processing and local reflexes. Inputs from the cervical area are involved in the cervico-collic reflex, the cervico-occular reflex and the tonic neck reflex, which provide information about the movement and position of the head in space that are crucial for both neck movement and postural control (16).

The cervical afferent input plays an important role to build up the internal reference frame for the control of posture and locomotion. Significant effects of head in space and head to trunk relation are observed in sensorimotor tasks (17, 18). If the input deteriorates or alters, the central nervous system (CNS) might increase the weighting of input from other locations (19). It is hypothesized that in healthy individuals, the preferred source of sensory inputs is somatosensory input from the feet in contact with the supporting surface (20). In contrast, older adults with CNP rely more on vision and other somatosensory inputs for postural control, and thus deficits will be greatest when these inputs are reduced (6–8). The modified Clinical Test of Sensory Integration on Balance (mCTSIB) is one of the most common clinical tools used in patients with postural control impairment to determine how well a patient uses the input from three sensory balance systems (somatosensory system, visual system, and vestibular system) during different balance activities. These activities include standing with eyes open/firm surface, eyes closed/firm surface, eyes open/soft surface, and eyes closed/soft surface (8, 21). However, mCTSIB with an adjusted base of support (mCTSIB-aBoS), including comfortable and narrow stance, has been used in previous studies to challenge the postural control system in older adults with CNP (21, 22). The tandem stance was excluded due to difficulty even in healthy older adults (23). The results showed that older adults with CNP demonstrated poorer postural control than healthy controls across sensorimotor integration tasks by increasing postural sway in the anteroposterior direction during the comfortable stance with eyes closed on a firm surface and eyes open on a soft surface and increasing postural sway in the mediolateral direction during the narrow stance with eyes open on a firm surface (21). Previous studies in older adults with CNP also reported a slower self-selected gait speed and cadence during the Timed Up and Go (TUG) test and the Ten Meter Walk (TMW) test with head turn condition, in addition to demonstrating a longer gait cycle duration in the TMW test both with and without head turns (6, 21). Furthermore, the studies demonstrated worse scores on the Dynamic Gait Index (DGI) in older adults with CNP than in healthy controls (6, 7). These problems may alter their functional balance, leading to restriction of walking or limited social participation and falls (6–8, 21).

Although impaired sensory integration is evident in older adults with CNP, other systems for postural control, i.e., the musculoskeletal system, internal representations, adaptive mechanisms, anticipatory mechanisms and neuromuscular synergies, have not been thoroughly investigated. Therefore, the extent of postural control impairments in older adults with CNP remains unclear. The Balance Evaluation Systems Test (BESTest) was developed based on the postural control system and was constructed to be a comprehensive balance measure in clinical settings for mixed populations (24). Six domains underlying the postural control system, biomechanical constraints, stability limits, transitions–anticipatory postural adjustment, reactive postural response, sensory orientation, and stability in gait, are included in the BESTest (24). The advantage of the BESTest is that it covers almost all systems underlying postural control so that clinicians can determine the types of balance training that are specific to the causes of postural control problems. The BESTest has been shown to be a reliable and valid measure of balance components in individuals with neurological disease (e.g., Parkinson’s disease, multiple sclerosis, and stroke). The BESTest can also be used to detect the function of the postural system in healthy individuals, which starts to decline as early as in the middle age group (41–60 years) (25). Furthermore, the BESTest can be used to discriminate between high vs. low risk of falls in adults aged 50 years and older (26). However, evidence of its use in older adults with CNP has not been reported.

Assessment of all postural control domains, as in the BESTest, could lead to early detection of balance impairment in older adults with CNP, so the specific intervention for improving balance can be promptly implemented. Therefore, this study aimed to investigate the use of the BESTest in older adults with CNP compared to older adults without CNP using young adults as the reference. We hypothesized that the BESTest would be able to identify system-specific postural control impairments in older adults with CNP. Rasch analysis (partial credit model) could provide valuable information related to item difficulty to determine the progression of balance exercises from easy to more difficult stages (27). In addition, this study revealed the level of BESTest item difficulty for older adults with and without CNP for further use in balance rehabilitation and fall prevention purposes.

## Materials and methods

### Participants

The sample size was determined based on a prior study (6), which showed an effect size of 0.52 between older adults with and without CNP and was used to estimate the sample size for this study. A power analysis performed with G*Power version 3.1.9.4 indicated that at least 15 participants in each group would be needed to ensure an adequate power level of 0.80 for the Kruskal–Wallis test at an alpha level of 0.05. Participants from three groups of subjects, healthy young participants aged 20–40 years and older adults aged 60 years or older with and without CNP were included in the study through a method of convenience sampling. All participants were able to walk independently. Neck pain was defined as pain and stiffness in the neck with or without radiating pain. To be eligible for the CNP group, participants had to suffer neck pain with an average weekly intensity of at least 3 cm on the 10 cm Visual Analog Scale (VAS) as a predominant complaint for at least 3 months.

Participants were excluded if they had major comorbidities that could affect balance measurements based on the following criteria: a previous history of neck and head trauma, recent orthopedic surgery or fracture (within the last 6 months), recent acute musculoskeletal injury or inflammatory joint disease/arthritis that required active management, known or suspected vestibular pathology, vertigo or dizziness from ear or brain disorders, neurological conditions, systemic conditions, use of medication that could affect balance, and cognitive impairment [as measured by the Montreal Cognitive Assessment (MoCA) with a total score of less than 24/30]. Ethical approval for the study was granted by the Human Research Ethics Committee of Srinakharinwirot University (SWUEC-039/2562F). Written informed consent was obtained before participation.

### Measurement tools

Several clinical scales were administered in this study. The demographic data of each participant were obtained *via* interviews and medical records. Age, sex, and body mass index (BMI) were collected from all participants. The medication intake, comorbidities, self-rated neck pain and disability, self-perceived handicap associated with dizziness, and balance confidence in daily activities were obtained from older adults with and without neck pain.

#### Questionnaires

Neck pain intensity was assessed as “pain at the moment” on a blank 10 cm visual analog scale (VAS), on which 0 cm corresponds to “no pain at all” and 10 cm corresponds to “worst imaginable pain.”

The Neck Disability Index (NDI) Thai version (28) was administered *via* an interviewer-assisted questionnaire to assess the degree of self-reported neck pain and disability. It consists of 10 items concerning daily living, pain and concentration. Each item is scored from 0–5, with 0 representing no disability and 5 signifying extreme disability, giving a total score of 50 or 100 percent. The total scores can be interpreted into the following 5 levels of disability in performing activities of daily living: 0–8%, no disability; 10–28%, mild disability; 30–48%, moderate disability; 50–64%, severe disability; and 70–100%, complete disability (29).

The Activities-specific Balance Confidence scale (ABC) was used to assess participants’ balance confidence. The ABC requires patients to indicate their confidence in performing 16 activities without losing their balance or becoming unsteady on an 11-point scale (0–100%). Each item describes a specific activity that requires progressively increased balance control. Greater scores indicate higher balance confidence.

The Dizziness Handicap Index (DHI) was used to examine the self-perceived handicap associated with dizziness. The DHI consists of 25 items divided into three subscales: physical, functional, and emotional. Higher scores indicate the maximum perceived disability, with a maximal score of 100. The DHI can be used to classify individuals into 3 levels of disability; a total score of 0–30 indicates mild disability, 31–60 indicates moderate disability, and 61–100 indicates severe disability (30).

#### Clinical balance tool

The participants were instructed to perform 27 tasks of the Balance Evaluation Systems test (BESTest) for a total of 36 items, as some items consist of 2–4 subitems (e.g., for left and right sides). Each item is scored on a 4-level ordinal scale from 0 (worst performance) to 3 (best performance). The scores are summed to obtain a total score out of a possible maximum score of 108 points. Scores for the total test, as well as for each section, are expressed as a percentage of total points (24).

### Procedures

After obtaining informed consent, demographic data were gathered by rater 1. The BESTest was administered in a quiet laboratory setting by rater 2, who was blinded to the demographic data and participant groups. The intrarater reliability of rater 2 for using the BESTest was calculated in 10 older participants using an intraclass correlation coefficient (ICC). The results showed that the intrarater reliability of rater 2 was high (ICC = 0.96).

The testing items were grouped into 6 sequences, which were initiated with different sections of the BESTest and followed by the subsequent sections. For example, in the 1st sequence, Section I of the BESTest was administered first, followed by Sections II, III, IV, V, and VI, and in the 2nd sequence, Section II was administered first, followed by Sections III, IV, V, VI, and I. The participants were randomly assigned into each sequence, and the researcher ensured that there was an equal number of participants in each sequence. Participants were encouraged to rest for 5 min as needed between each section of the test to avoid fatigue. The total testing time was approximately 2 h, but if the test could not be completed within 1 day, it was continued the next day. To verify the accuracy of the scoring, the entire testing session of each participant was videotaped for subsequent review.

### Statistical analysis

Descriptive statistics were used to describe demographic data. To compare the percentage of the BESTest total and each section score between three groups, the Kruskal–Wallis test was selected. The pairwise comparison was used to pinpoint the difference between group. The Mann–Whitney U test was used to compare the BESTest item scores between older adults with and without CNP in the selected BESTest section. The significance level was set to 0.05 for all tests.

Once the BESTest domains that were significantly different between older adults with and without CNP had been identified, receiver operating characteristic (ROC) curve analysis was further conducted on those BESTest domains to differentiate the older adults whose daily life had been affected by neck problems using the NDI scores as a reference: participants with disability (total score ≥ 10%) and without disability (total score < 10%). The area under the curve (AUC) and the specificity, sensitivity, and cutoff points were calculated. An AUC value of 0.7–0.9 is generally considered to be acceptable for differentiation (31). The largest Youden index (sensitivity + [1 − specificity]) was chosen as the cutoff score. Positive likelihood ratios (+LR) were calculated as sensitivity/(1 − specificity). Negative likelihood ratios (−LR) were calculated as (1 − sensitivity)/specificity. The greater the +LR is than 1.0, the more valuable the positive test result. The −LR indicates the usefulness of a negative test result: the greater the value is less than 1.0, the more valuable the negative test result (32). Posttest accuracy was later calculated from the proportion of true positives and true negatives in all tested cases.

The item difficulty measure was estimated from the BESTest item score of each participant group by Rasch analysis (partial credit model) (27) using WINSTEPS software 5.2.2 (Winsteps^®^, Portland, OR, USA). The “simulate data” option was used to strengthening the findings due to the small sample size. The item difficulty was expressed in a logit scale, in which the highest logit represents the most difficult item, and the lowest logit represents the easiest item.

## Results

One hundred and ten participants from three groups of subjects, 30 healthy young participants aged 20–40 years and eighty older adults aged 60 years or older with (*n* = 20) and without CNP (*n* = 60), were included in the study. The demographic data of the young adults (YOUNG), older adults without chronic neck pain (OLD) and older adults with chronic neck pain (CNP) are presented in Table 1. As expected, there were significant differences in age between young and older adults (*p* < 0.05), whereas older adults with and without CNP did not significantly differ in age or comorbidities. Most of the participants in all groups were female, without a significant difference in body mass index. The CNP group had moderate pain and none to mild disability of daily living affected by neck problems (from the NDI score) and were significantly worse than the OLD group (*p* < 0.05). Moreover, those with CNP had less balance confidence in performing daily activities than those without CNP (*p* < 0.05).

**TABLE 1:** Characteristic of participants.

	Young (*n* = 30)	Old (*n* = 60)	CNP (*n* = 20)
Age (years, mean ± SD)	24.20 ± 4.13	64.70 ± 3.74^†^	63.85 ± 3.73^‡^
Gender [female, n (%)]	21 (70.00)	46 (76.67)	16 (80.00)
BMI (kg/m^2^, mean ± SD)	22.14 ± 2.25	23.56 ± 2.97	23.65 ± 3.75
NDI (0–100, mean ± SD)	–	0.63 ± 1.35	13.63 ± 6.74*
ABC scale (%, mean ± SD)	–	94.08 ± 5.79	88.78 ± 10.67*
DHI (points, mean ± SD)	–	0.03 ± 0.26	1.45 ± 4.51*
VAS (0–100, mean ± SD)	–	–	4.50 ± 1.47
Duration of neck pain (months, mean ± SD)	–	–	14.63 ± 14.15
**Side of neck pain [sides, n (%)]**			
- Right side	–	–	4 (20.00)
- Left side	–	–	4 (20.00)
- Both side	–	–	12 (60.00)
Comorbidities [conditions, median (SE)]	–	1.00 (0.71)	1.00 (0.63)
Taking more than four medications [n (%)]	–	5 (8)	2 (10)

NDI, Neck Disability Index; ABC, Activities-specific Balance Confidence scale; DHI, Dizziness Handicap Index; VAS, Visual Analog Scale; OLD, Older Adults without Chronic Neck Pain; YOUNG, Young Adults; CNP, Older Adults with Chronic Neck Pain.

^†^*p* < 0.05 comparison of YOUNG and OLD.

^‡^*p* < 0.05 comparison of YOUNG and CNP.

*p < 0.05 comparison of OLD and CNP.

The BESTest scores from three groups of participants, young adults and older adults with and without CNP, are presented in Table 2. Older adults with and without CNP demonstrated significantly lower BESTest total scores than young subjects. The comparison between the two groups of older adults showed that the CNP group had a lower BESTest total score than the OLD group. Regarding the section scores, the OLD group had a significantly lower score than the YOUNG group in all sections, except Section I (Biomechanical Constraints) and Section V (Sensory Integration), while the CNP group had lower scores than the YOUNG group in all sections except Section V. In addition, the CNP group had a significantly lower score than the OLD group in three sections: Biomechanical Constraints (Section I), Transitions–Anticipatory Postural Adjustment (Section III), and Reactive Postural Response (Section IV), which is 93.67 ± 5.91, 94.44 ± 6.74, and 89.17 ± 12.29, respectively (*p* < 0.05). Therefore, scores from these three sections (I, III, and IV) were selected for the following analyses.

**TABLE 2:** Percentage score in total and each section of the BESTest.

BESTest	Young (*n* = 30)	Old (*n* = 60)	CNP (*n* = 20)
Total (%)	99.73 ± 0.64	94.26 ± 3.35^†^	91.58 ± 3.11^‡^^*^
Section I: Biomechanical constraints (%)	99.33 ± 0.06	97.56 ± 4.42	93.67 ± 5.91^‡^^*^
Section II: Stability Limits/Verticality (%)	100.00 ± 0.00	88.97 ± 6.88^†^	89.29 ± 8.30^‡^
Section III: Transitions-anticipatory postural adjustment (%)	100.00 ± 0.00	97.41 ± 4.95^†^	94.44 ± 6.74^‡^^*^
Section IV: Reactive postural response (%)	100.00 ± 0.00	95.56 ± 5.58^†^	89.17 ± 12.29^‡^^*^
Section V: Sensory orientation (%)	100.00 ± 0.00	99.56 ± 2.08	99.33 ± 2.05
Section VI: Stability in gait (%)	99.05 ± 0.48	86.51 ± 7.55^†^	83.57 ± 4.76^‡^

OLD, Older Adults without Chronic Neck Pain; YOUNG, Young Adults; CNP, Older Adults with Chronic Neck Pain.

^†^*p* < 0.05 comparison of YOUNG and OLD.

^‡^*p* < 0.05 comparison of YOUNG and CNP.

*p < 0.05 comparison of OLD and CNP.

The frequency distribution of the BESTest scores within Sections I, III, and IV between older adults with and without CNP are shown in Table 3. Compared to the OLD group, the CNP group demonstrated a lower percentage of individuals who scored “normal” (three scores), which differed significantly in the following items: Section I, hip/trunk lateral strength; Section III, stand on non-dominant leg; and Section IV, compensatory stepping correction–forward and backward.

**TABLE 3:** The frequency distribution of the BESTest scores in section I, III, IV.

BESTest	Frequency (%)			
	
	Old (*n* = 60)		CNP (*n* = 20)	
		
	Normal	Abnormal	Normal	Abnormal
**Section I: Biomechanical constraints**				
- Base of support	100	0.00	100	0.00
- COM alignment	100	0.00	100	0.00
- Ankle strength and range	78.3	21.7	60.0	40.0
- Hip/Trunk lateral strength	88.3*	11.7	60.0*	40.0
- Sit on floor and standup	100	0.00	100	0.00
**Section III: Transitions-anticipatory postural adjustment**				
- Sit to stand	100	0.00	100	0.00
- Rise to toes	88.3	11.7	90.0	10.0
- Stand on non-dominant leg	85.0*	15.0	55.0*	45.0
- Stand on dominant leg	88.3	11.7	75.0	25.0
- Alternate stair touching	100	0.00	100	0.00
- Standing arm raise	100	0.00	100	0.00
**Section IV: Reactive postural response**				
- In place response: Forward	100	0.00	95.0	5.00
- In place response: Backward	93.3	6.70	85.0	15.0
- Compensatory stepping correction: Forward	96.7*	3.33	75.0*	25.0
- Compensatory stepping correction: Backward	90.0*	10.0	60.0*	40.0
- Compensatory stepping correction: Lateral (Non-dominant)	73.3	26.7	60.0	40.0
- Compensatory stepping correction: Lateral (Dominant)	71.7	28.3	60.0	40.0

OLD, Older Adults without Chronic Neck Pain; CNP, Older Adults with Chronic Neck Pain; Normal, Able to perform the test perfectly and score as 3; Abnormal, Unable to perform the test perfectly and score as 2, 1, or 0. Percentage of frequency was calculated by dividing the amount of participant in each score by the total participants of each group, and then multiplying the result by 100.

*p < 0.05 comparison of OLD and CNP.

Findings from the ROC analysis on the summative scores from Sections I, III and IV are shown in Table 4. The AUC was 0.79, indicating good diagnostic accuracy for classifying older adults with mild disability from neck pain, with a cutoff score of 48.5 out of 51. The sensitivity and specificity were high (72 and 69%, respectively), with acceptable LRs and good posttest accuracy (71.25%).

**TABLE 4:** Cutoff points for the summation score of section I, III, and IV from the BESTest with associated area under the curve of receiver operating characteristic curve, sensitivity and specificity, and likelihood ratios in older adults with and without disability (*N* = 80).

Variables	Total score of section I, III, and IV
Area under the curve	0.79
Cutoff score (/51)	48.5
Sensitivity	0.72
Specificity	0.69
Positive likelihood ratio	2.32
Negative likelihood ratio	0.41
Accuracy (%)	71.25%

Closer examination of each BESTest item difficulty level of older adults with and without CNP is presented in Tables 5, 6, respectively. The item order was determined by its difficulty from the easiest to the most difficult. All items of the BESTest were found to be too easy for young adults (item difficulty = −7.54, standard error = 2.04), except one item, the Timed Up and Go with dual task test, which was the most difficult item. Similarly, the Timed Up and Go with dual task item was also found to be the most difficult item for older adults with and without CNP. In contrast, eleven items were found to be the easiest items for both older adults with and without CNP, including base of support, center of mass alignment, sit on floor and standup, sit to stand, alternate stair touching, standing arm raise, sensory integration for balance—eyes open on firm surface, eyes closed on firm surface, eyes open on soft surface, and incline–eyes closed—and walk with pivot turns. Apart from these similarities, hip/trunk lateral strength, stand on non-dominant leg, and compensatory stepping correction–forward and backward were found to be harder for the CNP group than for the OLD group.

**TABLE 5:** Item difficulty measures of the BESTest in older adults with chronic neck pain (CNP).

Items	Item difficulty	Standard error (SE)	Items	Item difficulty	Standard error (SE)
Base of support	−3.34	1.84	Walk with head turns—Horizontal	–0.87	0.63
COM alignment	−3.34	1.84	In place response—Backward	–0.87	0.63
Sit on floor and standup	−3.34	1.84	Sitting lateral lean to non-dominant side	–0.87	0.63
Sit to stand	−3.34	1.84	Sitting verticality (Dominant side)	–0.23	0.51
Alternate stair touching	−3.34	1.84	Compensatory stepping correction Forward	0.02	0.48
Standing arm raise	−3.34	1.84	Stand on dominant leg	0.02	0.48
Sensory integration for balance - Eyes open on firm surface	−3.34	1.84	Compensatory stepping correction - Backward	0.43	0.44
Sensory integration for balance - Eyes closed on firm surface	−3.34	1.84	Ankle strength and range	0.43	0.44
Sensory integration for balance - Eyes open on foam surface	−3.34	1.84	Compensatory stepping correction - Lateral (Dominant side)	0.79	0.41
Sensory integration for balance Incline-eyes closed	−3.34	1.84	Compensatory stepping correction Lateral (Non-dominant side)	0.96	0.40
Walk with pivot turns	−3.34	1.84	Functional reach lateral (Dominant side)	0.96	0.40
Step over obstacles	−2.10	1.03	Functional reach forward	0.96	0.40
Change in gait speed	−2.10	1.03	Hip/Trunk lateral strength	0.96	0.40
In place response—Forward	−2.10	1.03	Stand on non-dominant leg	1.12	0.39
Sitting lateral lean-to dominant side	−2.10	1.03	Functional reach lateral (Non-dominant side)	1.12	0.39
Sensory integration for balance - Eyes closed, foam surface	−1.34	0.75	Gait—Level surface	1.56	0.38
Rise to toes	−1.34	0.75	Timed “Get Up and Go”	1.84	0.37
Sitting verticality (Non-dominant side)	−1.34	0.75	Timed “Get Up and Go” with dual task	4.08	0.44

COM, Centre of Mass.

**TABLE 6:** Item difficulty measures of the BESTest in older adults without chronic neck pain (OLD).

Items	Item difficulty	Standard error (SE)	Items	Item difficulty	Standard error (SE)
Base of support	−4.22	1.84	Sitting lateral lean to dominant side	–0.95	0.46
COM alignment	−4.22	1.84	Sitting verticality (Dominant side)	–0.95	0.46
Sit on floor and standup	−4.22	1.84	Compensatory stepping correction - Backward	–0.95	0.46
Sit to stand	−4.22	1.84	Rise to toes	–0.75	0.43
Alternate stair touching	−4.22	1.84	Sitting lateral lean to non-dominant side	–0.58	0.40
Standing arm raise	−4.22	1.84	Hip/Trunk lateral strength	–0.42	0.39
Sensory integration for balance - Eyes open on firm surface	−4.22	1.84	Sitting verticality (Non-dominant side)	–0.28	0.37
Sensory integration for balance - Eyes closed on firm surface	−4.22	1.84	Stand on non-dominant leg	–0.28	0.37
Sensory integration for balance - Eyes open on foam surface	−4.22	1.84	Stand on dominant leg	–0.14	0.36
Sensory integration for balance - Incline-eyes closed	−4.22	1.84	Ankle strength and range	0.10	0.34
Walk with pivot turns	−4.22	1.84	Compensatory stepping correction - Lateral (Non-dominant side)	0.50	0.31
In place response—Forward	−4.22	1.84	Compensatory stepping correction - Lateral (Dominant side)	0.68	0.29
Compensatory stepping correction - Forward	−2.23	0.74	Functional reach forward	1.38	0.36
Change in gait speed	−2.23	0.74	Gait—Level surface	1.58	0.26
Step over obstacles	−1.77	0.62	Functional reach lateral (Dominant side)	1.97	0.25
In place response—Backward	−1.44	0.54	Timed “Get Up and Go”	2.22	0.25
Sensory integration for balance - Eyes closed, foam surface	−1.44	0.54	Functional reach lateral (Non-dominant side)	2.40	0.25
Walk with head turns—Horizontal	−1.44	0.54	Timed “Get Up and Go” with dual task	4.08	0.44

COM, Centre of Mass.

## Discussion

The BESTest is a comprehensive clinical tool for balance measurement based on the conceptual model of balance control in which 6 different systems contribute to the control of balance and posture. This study is the first to investigate the use of the BESTest in older adults with CNP to identify which system of balance control would be impaired as a result of CNP. The OLD and YOUNG groups were also investigated in this study to control for the effect of confounding age factors. Corresponding to the study’s hypothesis, our results demonstrated that the BESTest can be used to identify system-specific postural control impairments in CNP. The BESTest scores showed that balance control was deteriorated from the normal aging process and further declined in the CNP group, such that CNP affected three balance control systems, biomechanical constraints, transitions–anticipatory postural adjustment, and reactive postural response, when compared with the OLD group.

Biomechanical constraints correspond to the musculoskeletal system, including muscle strength, range of motion and body alignment. In contrast to a previous study (33), this study demonstrated no significant differences between the OLD and YOUNG groups. This disagreement could be due to different participant characteristics; those in our OLD group were younger and a high level of physical functioning, as indicated by an ABC score of more than 80 (34). However, the problem with biomechanical constraints was found to be declining in the CNP group. Closer examination (Table 3) showed that decreased hip/trunk lateral strength in the CNP group is a major problem. According to previous studies, CNP was found to increase concerns about falling and decrease physical performance (5), whereas hip muscle strength was reported to be an important indicator of physical performance, especially in elderly women (35). This finding was associated with the results of transitions–anticipatory postural adjustment (Section III), which was found to decline from the aging process and further declined when an individual experienced CNP. Standing on the non-dominant leg was the item from Section III that showed a significant difference between the OLD and CNP groups. A previous study (36) showed that vibratory stimulation directed to the dorsal neck muscles in human perturbed proprioceptive information and led to postural control instability during standing, suggesting that cervical afferent inputs play a dominant role in postural control in an upright stance. Altered cervical afferent inputs can be caused by CNP from a pain-induced change in nociceptor and mechanoreceptor activity at the spinal cord and within the CNS (12) or from chemical changes caused by inflammatory events that affect the sensitivity of the receptors (13). Other factors involve awkward postures, static and repetitive work, or trauma that disturbs the sensitivity of the cervical joint and muscle receptors (15). Thus, disturbed lower extremity muscle activity by altered cervical afferent inputs combined with decreased hip/trunk lateral strength from declining physical performance in individuals with CNP can affect their balance control.

There was a greater deficit in the reactive postural response in older adults with CNP than in those with normal aging, suggesting that most of them had failed to preserve postural stability by activating the stepping strategy. Compared to the OLD group, a higher number of older adults with CNP had significant problems with compensatory stepping correction in both forward (25%) and backward directions (40%), where participants were asked to stand with feet shoulder width apart, arms at their sides and lean forward/backward against the researcher’s hands until their shoulders and hips were out of line with their toes and the researcher suddenly released the support to elicit the step. The central nervous system (CNS) is responsible for integrating afferent inputs and sending postural adjustments to maintain the center of gravity over the base of support. If somatosensory inputs are impaired, the CNS will be unable to select the correct strategies in time (37). The cervical spine has an important role in providing afferent inputs for the internal reference frame to maintain postural stability, since the main input comes from at least three sources, including somatosensory (local and distal), visual, and vestibular systems (20). Furthermore, cervical proprioceptors provide the CNS with information about the movement and location of the head in relation to the trunk. The cervical muscles, which have a high concentration of muscle spindles, relay information to and receive information from the CNS, and there are specific connections between the cervical receptors, the visual and vestibular apparatus and the autonomic nervous system (38). Cervical proprioceptors are involved in the cervico-collic reflex, the cervico-occular reflex and the tonic neck reflex, which provide information about the movement and position of the head in space (16). Older adults with CNP demonstrated sensorimotor disturbances caused by altered cervical afferent inputs in terms of greater deficits in eye movement control, vertical perception, and postural control (6–8, 21). Therefore, impairments in sensorimotor integration caused by CNP may lead to impaired reactive postural responses. In addition, our study demonstrated a trend for those with CNP to have problems with the compensatory stepping correction in backward directions more than forward directions when compared to the OLD group. Backward stepping requires more effort than forward stepping since the margin of stability is smaller and there is greater instability in the backward direction (39). Furthermore, aging was found to affect the recruitment of proper muscle synergies during reactive backward stepping. Changes in the contribution of tibialis anterior, biceps femoris (long head) and gastrocnemius muscles in the stance limb of older adults may contribute to decrease in step length during reactive backward stepping when compared to young adults (40). Although no differences between the OLD and CNP groups were found during the compensatory stepping correction on either lateral side, both groups demonstrated lower scores than the YOUNG group. Thus, compensatory stepping correction in all directions needs to be considered in CNP.

This study demonstrates that aging has a deteriorating effect on multiple aspects of postural control, except sensory orientation. Our result was not in accordance with previous studies that reported a significant difference in the sensory integration declined by both aging and CNP (6–8, 21, 41). The discrepancy of findings may be because the tasks and measurement tools are not entirely comparable. In this study, the participants were examined by a clinical tool (BESTest) that included the mCTSIB and standing balance test with eyes closed on an inclined surface to determine sensory integration without using laboratory tools, whereas in previous studies (6–8, 21, 41), the participants were examined by various tests using laboratory tools. Furthermore, Rasch analysis showed that all items in Section V (Sensory Orientation) of the BESTest were the easiest items. Thus, the BESTest alone might not be suitable for clinically examining sensory integration in those who have a high level of physical functioning.

In this study, stability in gait scores (Section VI) in both older adult groups were significantly lower than those in the YOUNG group, but we did not find a section score difference between the OLD and CNP groups. Our findings do not agree with those of previous studies (6, 8, 21), which found slower walking speed during the Timed Up and Go (TUG) test, poorer scores on the Dynamic Gait Index (DGI), and gait parameter disturbance during the Timed Ten Meter Walk test with and without head movement in CNP. This may be caused by the differences in age and disability level caused by neck problems, where participants in the previous study were older and had moderate disability. However, according to the results of the Rasch analysis of the BESTest, gait assessment was more challenging for the CNP group than for the OLD group. The Timed Up and Go with dual task test was found to be the most difficult item for the CNP group, followed by the TUG and gait–level surface tests, which may be attributed to both cognitive decline of normal aging and impaired balance control from CNP. Most participants in the OLD (67–85%) and CNP (85–90%) groups were unable to complete walking 20 feet on an even surface within 5.5 s and TUG within 11 s. Gait speed is an essential component for identifying a history of falls (42), and the TUG test alone is a sensitive and specific test for identifying risk factors for falls in older adults (43). The dual task used in the BESTest is a cognitive task (counting backward by threes from 100); when combined with the TUG test, it can be used to detect the risk of falls and mild cognitive impairment-related changes in older adults (44). Impairments in stability in gait combined with lower extremity muscle weakness and impaired balance should be a concern, since all are considered risk factors for falls (45).

The BESTest has been known for its long administration time such that it would take up to 35 min to complete the test. Our study demonstrated that not all BESTest domains were found to be deficit in older adults with chronic neck pain. Also, the sensory orientation domain of the BESTest was found too easy to perform for both older adults with and without chronic neck pain. To reduce the assessment time, this study proposed using the combined score from BESTest domains that were significantly different between older adults with and without CNP, as a screening test. Results revealed that the BESTest can be used in the detection of system-specific postural control impairments in older adults with CNP by using the total score of Sections I, III, and IV as a screening tool for differentiating older adults whose daily life had been affected by neck problems with a high AUC (0.79), sensitivity (72%), and specificity (69%). The BESTest also has a good posttest accuracy (71.25%) using the suggested cutoff score of 48.5 out of 51. The participants in the CNP group presented with relatively moderate levels of neck pain intensity (average pain intensity = 4.50/10) and mild neck disability (average NDI score = 13.63/100). Although the average ABC scale, which represented the fear of falling, was significantly lower in the CNP group than in the OLD group, the scores of the CNP group were relatively good and they were considered to have a high level of physical functioning (34) (average ABC scale = 88.78/100). This is relevant, as it highlights that decreased postural control as measured by the BESTest can be found in the CNP, even with relatively moderate pain, mild disability and a high level of physical functioning.

Our study has several implications. First, the BESTest can be used to assess system-specific postural control impairments in older adults with and without chronic neck pain, as the BESTest total scores were significantly different among three groups of participants (YOUNG, OLD, and CNP). However, clinicians should be aware that these differences, especially the score differences between OLD and CNP, may not be clinical significance, as their values may not reach minimal clinically important differences (MCID). Second, the results revealed that the BESTest can be used in the detection of system-specific postural control impairments in older adults with CNP by using the total score of Sections I, III, and IV as a screening tool. Third, the results suggested that older adults with CNP who have moderate pain and mild disability may have lower extremity muscle imbalance and a reduced ability to compensate for stepping correction, especially in the forward and backward directions. However, other balance problems can also be found, since significant differences were reported in almost all subsystems except sensory integration when compared to young adults. Impairments in stability in gait combined with lower extremity muscle weakness and impaired balance are considered risk factors for falls (45). Therapists need to be mindful of the balance problem caused by normal aging and CNP. We suggest that therapists administer Sections II and VI of the BESTest after finding a positive result by screening, with the total score of Sections I, III, and IV used to obtain complete information on postural control system impairment. Lastly, the hierarchical order of the item difficulty suggested that 11 out of 36 items of the BESTest do not challenge older adults with CNP who have moderate pain and mild disability. However, the remaining items can provide valuable information for therapists to implement specific training and determine the progression of balance training from easy to more difficult stages. For example, if a patient is unable to complete hip/trunk lateral strength and stand on one leg, it is recommended to start with hip/trunk muscle strengthening before progressing to standing on one leg.

The results of this study should be interpreted in light of its limitations. First, to control the effects of the aging process in the CNP group, older adults with any kind of pathology related to balance control were excluded from this study, resulting in the small sample size of individual with CNP. It is well-known that older adults with CNP in general have multiple health problems and complications; thus, a higher severity of balance problems could be expected. Nevertheless, individuals with CNP with other comorbidities typically found in older adults should be recruited in future studies to confirm this speculation. Second, most participants in the CNP group had moderate pain and mild disability. It is necessary to be concerned that the severity of the problem may vary with patients who have different levels of pain and disability. Third, the current study did not compare each of the BESTest domain score with specific measurements, such as muscle strength, endurance, EMG responses, rather this study used the performance of the older adults without CNP as the comparison for identifying system-specific balance impairments. Therefore, future studies with direct comparison of the BESTest score and other standardized tools are required to confirm the construct validity of the BESTest in older adults with CNP.

## Conclusion

The BESTest can be used to identify system-specific postural control impairments in older adults with CNP. The BESTest scores showed that balance control deteriorated from the normal aging process and further declined in CNP. Three sections of the BESTest, biomechanical constraints, transitions–anticipatory postural adjustment, and reactive postural response, were suggested for the detection of system-specific postural control impairments in older adults whose daily life was affected by neck problems. The Rasch analysis revealed 14 items of the BESTest that were difficult for older adults with CNP and could be further used for balance rehabilitation and fall prevention.

## Data availability statement

The raw data supporting the conclusions of this article will be made available by the authors, without undue reservation.

## Ethics statement

The studies involving human participants were reviewed and approved by Human Research Ethics Committee of Srinakharinwirot University (SWUEC-039/2562F). The patients/participants provided their written informed consent to participate in this study.

## Author contributions

RB conceived and designed the project, procured funding, analyzed the data, and prepared the final manuscript. TM prepared instrument, collected and analyzed the data, and wrote the first draft of the manuscript. TT helped with the data analysis. CC helped in the preparation of the final manuscript. All authors contributed to the article and approved the submitted version.
